# Metabolic rate and body size are linked with perception of temporal information^☆^

**DOI:** 10.1016/j.anbehav.2013.06.018

**Published:** 2013-10

**Authors:** Kevin Healy, Luke McNally, Graeme D. Ruxton, Natalie Cooper, Andrew L. Jackson

**Affiliations:** a Department of Zoology, School of Natural Sciences, Trinity College Dublin, Ireland; b Trinity Centre for Biodiversity Research, Trinity College Dublin, Ireland; c Centre for Immunity, Infection and Evolution, School of Biology, University of Edinburgh, Edinburgh, U.K.; d School of Biology, University of St Andrews, St Andrews, U.K.

**Keywords:** comparative analysis, critical flicker fusion, evolutionary ecology, predator–prey, temporal resolution

## Abstract

Body size and metabolic rate both fundamentally constrain how species interact with their environment, and hence ultimately affect their niche. While many mechanisms leading to these constraints have been explored, their effects on the resolution at which temporal information is perceived have been largely overlooked. The visual system acts as a gateway to the dynamic environment and the relative resolution at which organisms are able to acquire and process visual information is likely to restrict their ability to interact with events around them. As both smaller size and higher metabolic rates should facilitate rapid behavioural responses, we hypothesized that these traits would favour perception of temporal change over finer timescales. Using critical flicker fusion frequency, the lowest frequency of flashing at which a flickering light source is perceived as constant, as a measure of the maximum rate of temporal information processing in the visual system, we carried out a phylogenetic comparative analysis of a wide range of vertebrates that supported this hypothesis. Our results have implications for the evolution of signalling systems and predator–prey interactions, and, combined with the strong influence that both body mass and metabolism have on a species' ecological niche, suggest that time perception may constitute an important and overlooked dimension of niche differentiation.

## Highlights

• Animals vary in their ability to perceive changes in their environment visually.
• Temporal perception can be quantified using critical flicker fusion (CFF).
• High CFF indicates an ability to perceive rapid changes in the visual field.
• We show that high metabolism and small body size are associated with high CFF.
• We argue that these findings have both ecological and evolutionary implications.

All biological systems, from organisms to ecosystems, are shaped by universal constraints. For example, body size and metabolic rate act as important constraints on several characteristics of organisms such as life history and behaviour, making them a particularly common and well-studied aspect of species' ecology (Brown et al. 2004; Woodward et al. 2005; Sibly et al. 2012). However, constraints imposed by the organism's sensory limitations are probably equally important and yet frequently overlooked (McGill & Mittelbach 2006; Pawar et al. 2012).

**Figure 1:**
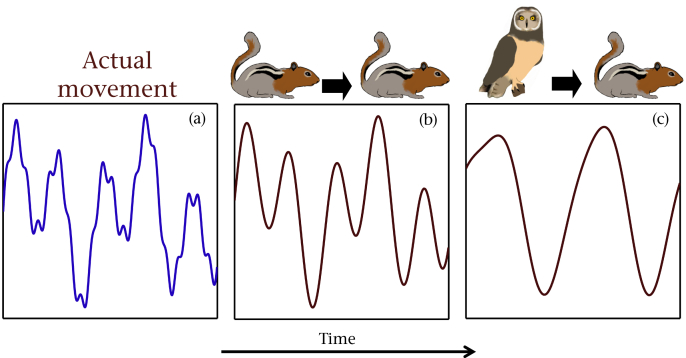
The ability of an organism to track a moving object depends on the time integral over which the individual can obtain its information. This is determined by its ability to resolve temporal information. In cases where an animal, such as a ground squirrel, displays complex movement (a), conspecifics may perceive the individual as moving according to a first-order integral of its actual movement owing to its high temporal resolution abilities (b). However a species with lower temporal resolution abilities, such as a short-eared owl, may perceive the motion as an even higher order derivative of the actual motion, meaning information of prey motion at finer temporal scales is not available to it (c).

In animal species, the limitations of sensory systems are crucial in shaping both intra- and interspecific interactions. For example the ability to spot and accurately predict the motion of the opposite party can be pivotal in determining the outcome in both predator–prey interactions (Fig. 1; Cronin 2005; Stevens 2007; Stevens et al. 2011; Clark et al. 2012; De Vries & Clandinin 2012) and the locating of mates (Land & Collett 1974; Hornstein et al. 2000). While the links among sensory limitations, foraging and spatial acuity have been studied in detail (e.g. in the use of search images for prey detection; Cronin 2005), the temporal resolution at which dynamic information can be perceived has received considerably less attention, in particular within a general ecological and evolutionary context.

The ability to integrate information over fine timescales, that is, at high temporal resolution, is thus fundamental to many aspects of an organism's ecology and behaviour. Furthermore, temporal resolution is also directly linked to the perception of the passage of time itself for humans, in particular when tracking fast moving stimuli (Hagura et al. 2012). From an evolutionary perspective, a trade-off exists between the demand for information at high temporal resolution and the costs of its acquisition given the energetic demands associated with increased rates of neural processing in the visual system (Laughlin 2001). This trade-off is likely to be shaped by various ecological (e.g. mode of predation) and environmental factors (e.g. light levels) as well as intrinsic factors (e.g. morphology) that will ultimately shape an organism's optimal temporal resolution for sensory perception. For example, predators of slow-moving prey may require less temporal resolution than predators that engage in active pursuit of fast-moving prey, such as raptors catching prey during flight.

This ability to perceive and react to a dynamic environment is a key behavioural and ecological trait. Ecologically, interaction strengths can be affected by the ability to identify and track fast-moving objects such as prey or mates (Fig. 1; Land & Collett 1974; Fritsches et al. 2005). The necessity of this ability to perceive one's environs accurately is perhaps best demonstrated in cases where temporal resolution is too coarse to allow the observer to follow the motion of a moving target accurately. A stark demonstration of this can be seen in the tiger beetle, *Cicindela hudsoni*, which, owing to the relatively low temporal resolution of its visual system, must take a stop–start approach in order to recalibrate the position of its prey when hunting (Gilbert 1997). In humans, the limitations of our temporal perception are apparent when tracking fast-moving objects such as the curving trajectory of a ball in soccer (Dessing & Craig 2010) and baseball (Bahill & Baldwin 2004).

Two intrinsic factors that may shape the costs and benefits of the temporal resolution of the sensory system, in particular with respect to their effects on an individual's ability to interact with the environment on short timescales, are body size and metabolic rate. As larger body sizes decrease manoeuvrability (Heglund & Taylor 1988; Dudley 2002; Biewener 2003; Sato et al. 2007; Vogel 2008; Hedrick 2011; Watanabe et al. 2012) and higher metabolic rates increase both manoeuvrability and the physiological ability to process information (Laughlin 2001; Franz & Ronacher 2002), we hypothesized that smaller organisms and those with higher metabolic rates perceive temporal change on finer timescales.

To quantify the temporal perceptual abilities of a range of species we took advantage of the all or nothing nature of neural firing in the visual system. Owing to this binary firing, temporal resolution must be encoded in terms of discrete units, as biological visual systems must discretize the continuous-time and continuous-space information reaching the retina and then integrate this information over some time period. This ‘integration time’ of visual systems can be quantified using the critical flicker fusion frequency (CFF): the lowest frequency of flashing at which a flickering light source is perceived as constant (D'Eath 1998; Schwartz 2009). As light intensity can increase the number of flashes that can be observed per second, the maximum CFF value, as measured in a response curve of CFF against light intensity (Ferry 1892; Porter 1902), can be used as a proxy for the temporal resolution of the sensory system.

We used CFF to compare the temporal resolution of the visual system in a wide range of vertebrate species including representatives from Mammalia, Reptilia, Aves, Amphibia, Elasmobranchii and Actinopterygii. Using phylogenetic comparative methods and controlling for the light levels each species typically experiences, we tested whether the temporal resolution of the sensory system increases with mass-specific metabolic rate and decreases with body mass.

## Methods

### Data Collection

To test our prediction that CFF increases with mass-specific metabolic rate and decreases with body size (when controlling for light levels), we collated data on CFF values of vertebrate species from the literature (Table 1). We only included values from studies that measured CFF using either behavioural or electroretinogram (ERG) procedures. In behavioural studies, CFF is measured through conditional training with the subject trained to respond to a change in its perception of a light flashing (D'Eath 1998; Rubene et al. 2010). For example, Lisney et al. (2011) conducted behavioural tests in domestic chickens, *Gallus gallus*, through choice experiments using flickering and nonflickering stimulus windows with choice of the correct stimulus rewarded with food. This is repeated over a range of light intensities and flicker frequencies until individuals can no longer distinguish between the stimuli. In ERG studies, a direct measurement of the electrical response of the retina in reaction to a flashing light source is used as a measure of CFF (D'Eath 1998; Schwartz 2009). As there may be further processing of temporal information after it reaches the retina that may cause behavioural studies to measure lower CFF values (D'Eath 1998), we included the experimental procedure used to measure CFF as a candidate covariate in our models. We also noted whether each study was a reliable measure of the maximum possible CFF. As maximum CFF is a function of many variables, such as light intensity, and not all studies reported a sufficient range of intensities, their reported CFF may not be the ‘true maximum’ possible. To ensure this did not affect our results we ran an additional analysis that included a term based on this assessment as a categorical covariate as part of our sensitivity analyses (see Appendix).

We used mean body masses (g) published in the literature and in databases including FishBase (Froese & Pauly 2012) and Animal Diversity Web (Myers et al. 2012) for each species as the measure of body size. For metabolic rates we used mass-specific resting metabolic rate as measured by oxygen consumption through ventilation in studies in which the subjects were fasted prior to the measurement. We converted these values to W/g using the conversion of 20 J/ml of oxygen consumption (Makarieva et al. 2008) to allow comparison among species. For ram-ventilation species (which require constant movement to force fluid over the respiratory organs), such as sharks and tuna, the resting metabolic rate was taken as the fitted line of oxygen consumption with swimming speed extrapolated to the intercept (swimming speed = 0 m/s; Table 1). To account for the possible effect of metabolic rate measured at different temperatures in ectothermic species, metabolic rate values were corrected to 25 °C using Q10 values, i.e. the fold change in metabolic rate over a temperature change of 10 °C, for reptiles, amphibians and fish (White et al. 2006). These corrections gave values of temperature-corrected mass-specific resting metabolic rates (qWg), for each species. Although body mass and mass-specific metabolic rate are expected to be correlated according to an exponent of 0.25 (Brown et al. 2004; Sibly et al. 2012), we included both terms as recommended by Freckleton (2009) instead of using residuals from a regression of body mass against mass-specific metabolic rate.

As there is a trade-off between sensitivity and movement perception owing to the requirement of longer integration times in low light conditions (Tansley 1957), as is seen in the different light response dynamics of rods and cones (Rubene et al. 2010), we included light levels within our analyses as a categorical variable based on the light conditions experienced by the species during normal activity (i.e. foraging). Species were categorized as inhabiting either high or low light conditions with diurnal terrestrial and nonturbid aquatic species coded as inhabiting high light level environments and nocturnal species coded as inhabiting low light levels. As the light levels of species that inhabit turbid waters are typically orders of magnitude lower than typical daylight levels (40–1000 lx; Ali & Klyne 1985; Palmer & Grant 2010; Kreysing et al. 2012) and the harp seal, *Pagophilus groenlandicus*, regularly forages at depths greater than 200 m (Folkow et al. 2004) where light levels are comparable to nocturnal light levels (Palmer & Grant 2010), we categorized these species as inhabiting low light level environments.

**Figure A1:**
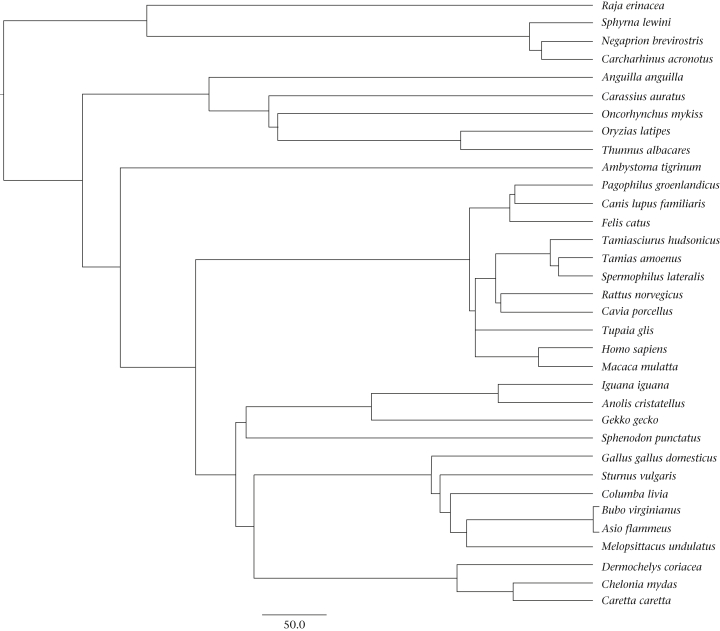
Species and phylogenetic relationship used in comparative analysis. Scale bar represent 50 million years. See Methods for details.

To correct for the phylogenetic nonindependence of species we constructed a composite tree of the study species using published molecular phylogenies and divergence times from various sources (Schoch 1985; Janossy 1986; Mercer & Roth 2003; Hedges et al. 2006; Wiens et al. 2006; Benton & Donoghue 2007; Murphy et al. 2007; Brown et al. 2008; Li et al. 2008; Naro-Maciel et al. 2008; Albert et al. 2009; Lim et al. 2010; Little et al. 2010; Perelman et al. 2011; see the Appendix and Fig. A1). In instances in which a divergence time was not available for two species we used the conservatively estimated date of first appearance as the divergence time taken from the Paleobiology Database (Alroy et al. 2008).

As ectotherm metabolic rates vary with temperature, we performed a sensitivity analysis to test the effect of the temperature to which qWg was corrected to by rerunning the main analysis with qWg corrected to both 5 °C and 35 °C (see Appendix). We also carried out a supplemental analysis on a more restricted data set for species with available brain mass data to test for any possible effects of sensory tissue on maximum CFF values (see Appendix).

In total we collected data on maximum CFF, body mass, qWg and light environments for 34 species across the vertebrate classes Elasmobranchii, Actinopterygii, Aves, Amphibia, Reptilia and Mammalia, with further data on brain mass for 28 of these species (Table 1).

### Statistical Analyses

To test our hypothesis we used a phylogenetic generalized least-squared approach (PGLS) using the caper package (Orme et al. 2012) in R version 2.14.2 (R Development Core Team 2012). The PGLS approach is based on standard generalized least-squared models while also accounting for the nonindependence in the data caused by species' phylogenetic relationships by incorporating it through the error term structure (Pagel 1999; Rohlf 2001). This error term consists of a matrix of expected trait covariances calculated using the maximum likelihood estimate of lambda (λ), a multiplier of the off-diagonal elements of a phylogenetic variance–covariance matrix that best fits the data. When the data are structured according to a Brownian motion of trait evolution, λ = 1, whereas when the data have no phylogenetic dependency, then λ = 0 (Pagel 1999).

We ran PGLS models with maximum CFF as the response variable, and all combinations of the following explanatory variables: body mass, qWg, light level (high, low) and experimental procedure (ERG, behavioural) with brain mass and methodological optimality included in the sensitivity analysis (see Appendix). We did not include interactions, as there was no a priori reason to include them. We used the Akaike information criterion (AIC), which penalizes extra effective parameters to avoid overparameterized models, to select the minimum adequate model (Burnham & Anderson 2002).

## Results

**Figure A2:**
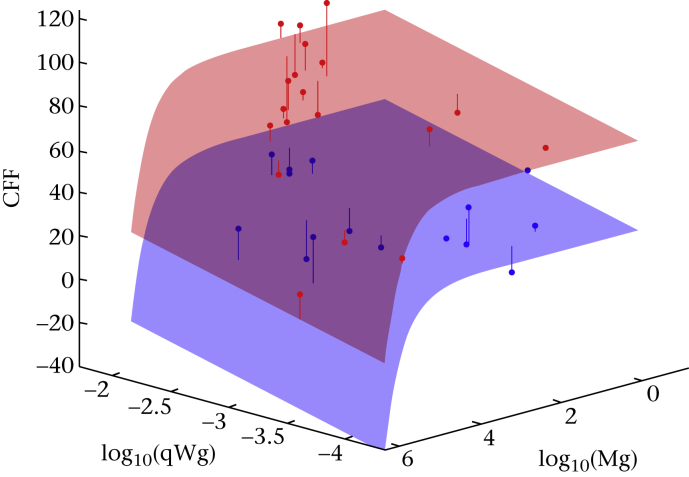
The effect of body mass (presented on log scale), light levels and log temperature-corrected mass-specific resting metabolic rate (qWg) on critical flicker fusion frequency (CFF). The minimal adequate model (Results) indicates CFF increases with log_10_ qWg (13.24 ± 4.08) but decreases with body mass (−0.0002 ± 0.00004). Low light levels (blue) are associated with low CFF values (−41.10 ± 4.96) in comparison to high light levels (red). Residual values for each species are shown for different light levels with stems connecting them to the model surface.

**Figure 2:**
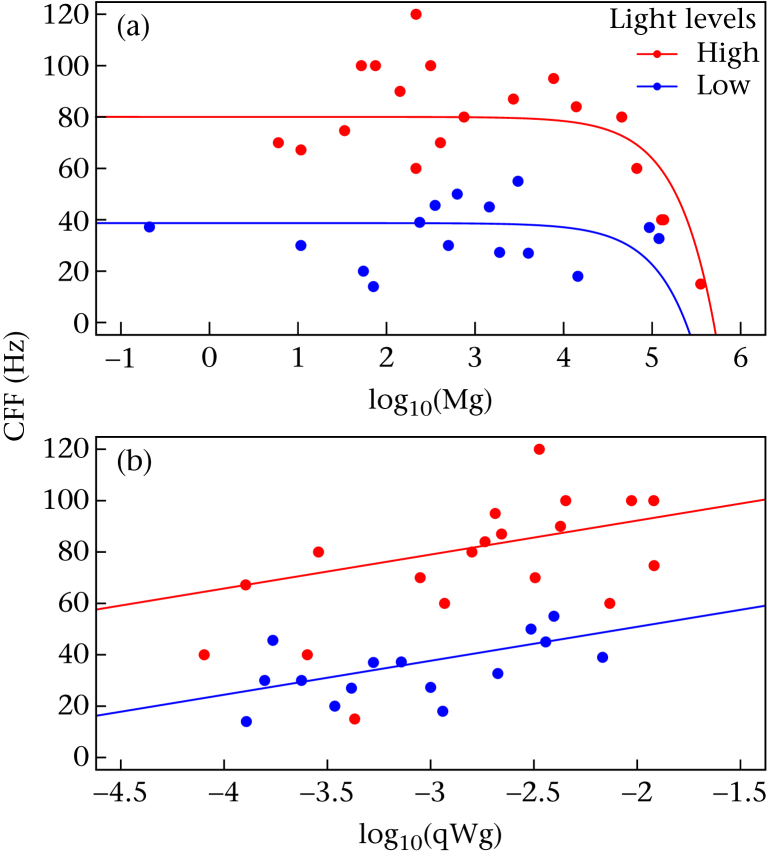
The effect of (a) body mass (presented on log_10_ scale) and (b) log_10_ temperature-corrected mass-specific resting metabolic rate (qWg) on critical flicker fusion frequency (CFF) while controlled for light levels. The minimal adequate model (Results) indicates CFF increases with log_10_ qWg (13.24 ± 4.08) but decreases with body mass (−0.0002 ± 0.00004). Low light levels are associated with low CFF values (−41.10 ± 4.96) in comparison to high light levels. Figure adjusted to display the intercept at the median value of the unrepresented axis.

The most parsimonious model (based on AIC) explaining variation in maximum CFF among vertebrates included the terms body mass, log_10_ of temperature-corrected mass-specific resting metabolic rate (qWg) and light level (Table 2, Tables A1 and A5 in the Appendix). The second most parsimonious model, which fell within two AIC values of the most parsimonious model, retained all tested variables (Table 2). Body mass had a negative effect on the temporal resolution of the sensory system (Table 2, Fig. 2a, Fig. A2 in the Appendix), with a change in body mass of approximately 10 kg resulting in a reduction in CFF of 2 Hz. The metabolic rate of organisms, after correcting for mass, was positively associated with CFF while low environmental light levels were associated with an overall reduction in CFF (Table 2, Fig. 2b, Fig. A2 in the Appendix). Phylogeny was found to have a minimal effect on the resulting models (λ = 0, Table 2) and experimental type was not correlated with CFF (Table 2). Thus, according to our model, small animals with high mass-specific metabolic rates in high light environments possessed the highest maximum CFF and hence greatest ability to perceive temporally dynamic visual information. Conversely, large animals with low mass-specific metabolic rates in low light environments had the lowest CFF.

These results were robust to our sensitivity analysis on both the temperature used to correct ectotherms qWg (taken as 25 °C in the main models above; see Methods) and the optimality of study methodology for measuring maximum CFF, with the best models in both sensitivity analyses (based on AIC) including the same terms and trends as found in the main analysis (Tables A2, A3, A5, A6, A7 and A9 in the Appendix). We also found that including brain mass in a restricted data set of 28 species for which brain mass was available did not change the effect of the explanatory variables light levels, qWg and body mass on maximum CFF (Tables A4 and A8 in the Appendix).

## Discussion

Many of the interspecific and intraspecific interactions that shape species' behaviour and ecology rely on the ability of organisms to process high temporal resolution sensory information. Our results show that, while there is considerable variability in the ability to resolve temporally dynamic visual information across vertebrates, body mass and metabolic rate act as important general constraints on this ability. This is the first study to indicate a general trend in the ability of vertebrates to resolve temporal information; previous studies have generally focused on specific cases of sensory adaptations (Fritsches et al. 2005) and particular environments (Frank 1999; Frank et al. 2012), hence focusing on the particular ecological context of each adaptation or environment. Our findings illustrate the relationship between both physiology and the effects of body mass on the ability to resolve temporal features of the environment on fine timescales, hence linking sensory adaptations to fundamental constraints and trade-offs imposed on all organisms.

Autrum's (1958) hypothesis, that the response dynamics of the retina should be shaped by the organism's particular ecology, predicts that organisms that demand fast visual systems will acquire adaptations increasing CFF values, and hence temporal resolution. For instance, given the strong effect of metabolic rate on CFF, one obvious adaptation is to alter the physiology and metabolism associated with the visual processing systems as seen in the localized heating of tissues in the heads of blowflies (Tatler et al. 2000) and the eyes of predatory swordfish (Fritsches et al. 2005). These tissues increase the temperature around the sensory tissues associated with the blowfly's or swordfish's visual system, which allows for an upregulation of CFF. Similar adaptations are also seen across species of large, fast-swimming predatory billfish (Carey 1982) and Lamnidae sharks (Block & Carey 1985). Physiological adaptations for high-resolution motion detection are also found within specific areas of the retina in some flies, commonly referred to as the ‘love spot’, which allow them to identify female flight patterns accurately and thus detect mates (Land & Collett 1974). Alterations to the rate of neuron firing, a fundamental limit to the rate of information transfer, through the provision of energy (Laughlin 2001) or changes in the physiological environment, as described above, would also allow for selection on temporal resolution abilities on a neurological level.

In a broader context, it might be expected that manoeuvrability, a vital component of an individual's ability to respond to the environment, may be one of the main factors determining whether it is necessary to invest in costly temporal information processing. Manoeuvrability, as defined by the ability to change body position or orientation, generally scales negatively with body mass. This negative scaling emerges primarily through the increased inertia and decreased limb stroke rate associated with large body size in both aquatic and volant species (Dudley 2002; Sato et al. 2007; Vogel 2008; Hedrick 2011; Watanabe et al. 2012), while in terrestrial species changes in gait posture that redistribute weight across the limbs can explain such reduced manoeuvrability with body mass (Heglund & Taylor 1988; Biewener 2003). These arguments show that, owing to the laws of physics, larger animals physically respond less quickly to a stimulus. Hence we expect selection against costly investment in sensory systems with unnecessarily high temporal resolution in large animals, as information on such timescales can no longer be utilized effectively. This may explain why larger vertebrates, along with those with low metabolic rates, had lower temporal resolution in our study. This idea is also supported by research showing that faster and more manoeuvrable fly species have higher temporal resolutions (Laughlin & Weckström 1993) and that less manoeuvrable scavenger crabs display slower response dynamics than deeper living predatory species which are likely to have more active lifestyles (Frank et al. 2012).

The effects of body size and metabolic rate on temporal resolution and the presence of sensory adaptations, as discussed above, also point towards an interesting dimension of niche space. Disparity in size and metabolic rate among species within an ecological setting may select for particular sets of adaptations creating a diverse set of sensory systems and interactions. In such a system, species might occupy the same spatial and temporal niche, but could be separated owing to differential responsiveness to environmental signals and cues as a result of having evolved divergent signalling systems along a dimension represented by temporal resolution. For example, it seems at least theoretically possible to encode information in high-frequency signals that can be detected by intended receivers such as conspecifics but that are not susceptible to ‘eavesdropping’ by (generally larger) predators. Ecological systems in which this may be apparent include deep-sea systems where visual signalling is an important determinant of the ability of organisms to interact, and where bioluminescence flashing over wide frequency ranges is ubiquitous (Haddock et al. 2005; Widder 2010).

In conclusion, our results show that the evolution of sensory systems, which play a vital role in ecological interactions, is subject to limitations imposed by metabolic rate and body mass over orders of magnitude in scale. Furthermore, deviations from the expected relationship between temporal perception, body size and metabolic rate are predicted to be subject to selection pressures for physiological, morphological and behavioural adaptations that alleviate these constraints. The generality of these findings suggest that temporal resolution may play a much more important role in sensory ecology than previously indicated, in particular because of its universal effects relating to body size. Further investigations into both the underlying mechanisms of these findings and their importance to ecological functioning are needed.

## Figures and Tables

**Figure A3:**
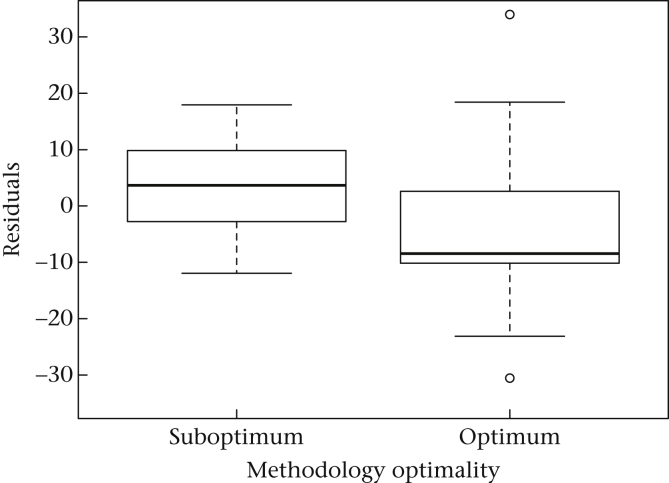
Residuals for optimum and suboptimum data quality taken from model 1 in main analysis. Box plot shows median (line), quartiles (box limits), 5th and 95th percentiles (error bars) and outliers (open circles).

**Figure A4:**
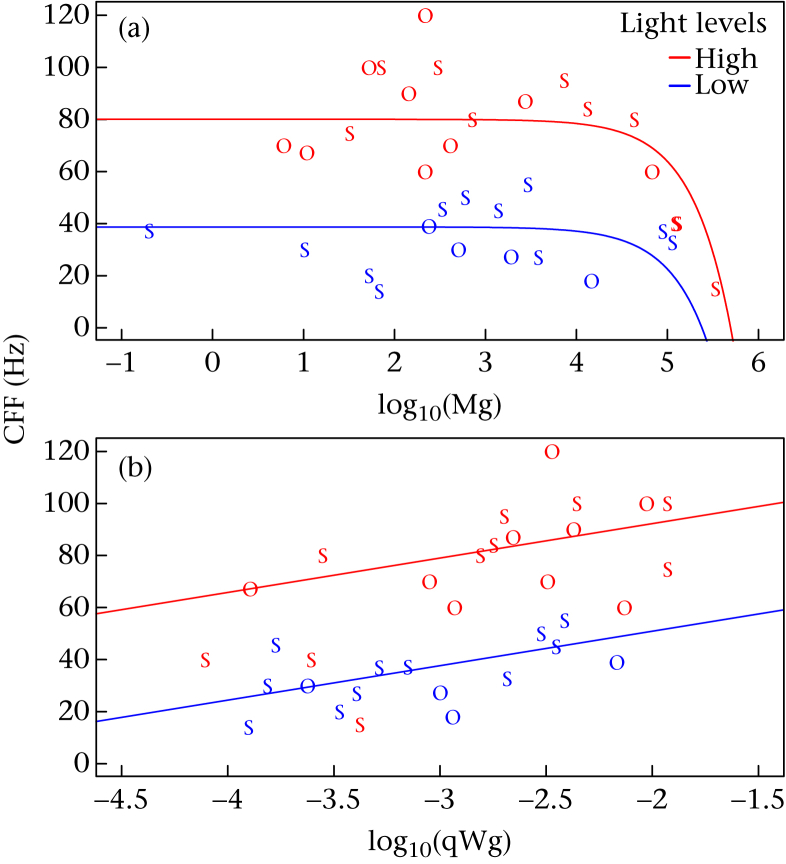
Plot of Fig. 1 with data quality represented with methodologically optimum (O) and methodologically suboptimum (S) data. Slopes corrected to represent the intercepts of each explanatory variable at the median value of (a) log_10_(qWg) and (b) log_10_ (Mg).

**Table A1:** Terms included in models performed in main analysis (see Methods)

Model	Explanatory variables in model				AIC	AICΔ
	Mg	log_10_(qWg)	Effect of light level	Experiment type		
1	+	+	+	−	275.70	0
2	+	+	+	+	277.68	1.98
3	+	−	+	−	283.94	8.24
4	+	−	+	+	285.91	10.21
5	−	+	+	−	291.56	15.86
6	−	+	+	+	293.41	17.71
7	−	−	+	−	298.90	23.20
8	−	−	+	+	300.63	24.93
9	−	+	−	−	315.06	39.36
10	−	+	−	−	315.07	39.37
11	−	+	−	+	316.84	41.14
12	+	+	−	+	316.92	41.22
13	+	−	−	−	320.67	44.97
14	+	−	−	+	322.67	46.97
15	−	−	−	+	324.45	48.75

Mg = body mass (g); qWg = temperature-corrected (25 °C) mass-specific resting metabolic rate W/g; AIC = Akaike's information criterion. AICΔ gives the difference between each model AIC and that of the lowest AIC found for any model. Terms retained are represented with + symbols, while terms not retained are represented by − symbols.

**Table A2:** Terms included in models performed in analysis with mass-specific resting metabolic rate qWg corrected to 5 °C

Model	Explanatory variables in model				AIC	AICΔ
	Mg	log_10_(qWg)	Effect of light level	Experiment type		
1	+	+	+	−	274.67	0
2	+	+	+	+	276.61	1.94
3	+	−	+	−	283.94	9.27
4	+	−	+	+	285.91	11.24
5	−	+	+	−	289.58	14.91
6	−	+	+	+	291.58	16.91
7	−	−	+	−	298.90	24.23
8	−	−	+	+	300.63	25.96
9	−	+	−	−	314.60	39.93
10	+	+	−	−	315.01	40.34
11	−	+	−	+	316.58	41.91
12	+	+	−	+	317.01	42.34
13	+	−	−	−	320.67	46.00
14	+	−	−	+	322.67	48.00
15	−	−	−	+	324.45	49.78

Mg = body mass (g); AIC = Akaike's information criterion. AICΔ gives the difference between each model AIC and that of the lowest AIC found for any model. Terms retained are represented with + symbols, while terms not retained are represented by − symbols.

**Table A3:** Terms included in models performed in analysis with mass-specific resting metabolic rate log_10_(qWg) corrected to 35 °C

Model	Explanatory variables in model				AIC	AICΔ
	Mg	log_10_(qWg)	Effect of light level	Experiment type		
1	+	+	+	−	277.39	0
2	+	+	+	+	279.26	1.87
3	+	−	+	−	283.94	6.55
4	+	−	+	+	285.91	8.52
5	−	+	+	−	294.26	16.87
6	−	+	+	+	295.79	18.40
7	−	−	+	−	298.90	21.51
8	−	−	+	+	300.63	23.24
9	+	+	−	−	315.97	38.58
10	−	+	−	−	316.49	39.10
11	+	+	−	+	317.53	40.14
12	−	+	−	+	317.89	40.50
13	+	−	−	−	320.67	43.28
14	+	−	−	+	322.67	45.28
15	−	−	−	+	324.45	47.06

Mg = body mass (g); AIC = Akaike's information criterion. AICΔ gives the difference between each model AIC and that of the lowest AIC found for any model. Terms retained are represented with + symbols, while terms not retained are represented by − symbols.

**Table A4:** Terms included in models performed in analysis including brain mass as a factor

Model	Explanatory variables in model					AIC	AICΔ
	Mg	log_10_(qWg)	Effect of light level	Brain mass	Experiment type		
1	+	+	+	−	−	219.56	0
2	+	+	+	−	+	221.27	1.71
3	+	+	+	+	−	221.54	1.98
4	+	+	+	+	+	223.26	3.70
5	+	−	+	−	−	226.27	6.71
6	+	−	+	+	−	227.99	8.43
7	+	−	+	−	+	228.24	8.68
8	+	−	+	+	+	229.94	10.38
9	−	+	+	−	−	234.55	14.99
10	−	+	+	+	−	235.91	16.35

Mg = body mass (g); qWg = temperature-corrected (25 °C) mass-specific resting metabolic rate W/g; AIC = Akaike's information criterion. AICΔ gives the difference between each model AIC and that of the lowest AIC found for any model. Terms retained are represented with + symbols, while terms not retained are represented by − symbols.

**Table A5:** Terms included in models performed in analysis with mass-specific resting metabolic rate qWg corrected to 35 °C

Model	Explanatory variables in model				AIC	AICΔ
	Mg	log_10_(qWg)	Effect of light level	Methodology		
1	+	+	+	+	275.05	0
2	+	+	+	−	275.80	0.75
3	+	−	+	+	283.74	8.69
4	+	−	+	−	283.90	8.85
5	−	+	+	−	291.74	16.69
6	−	+	+	+	293.69	18.64
7	−	−	+	−	298.86	23.81
8	−	−	+	+	300.39	25.34
9	−	+	−	−	315.35	40.30
10	+	+	−	−	315.37	40.32
11	+	+	−	+	317.04	41.99
12	+	+	−	+	317.29	42.24
13	+	−	−	−	320.92	45.87
14	+	−	−	+	322.26	47.21
15	−	−	−	+	323.38	48.33

Mg = body mass (g); methodology = optimality of study methodology; AIC = Akaike's information criterion. AICΔ gives the difference between each model AIC and that of the lowest AIC found for any model. Terms retained are represented with + symbols, while terms not retained are represented by − symbols.

**Table A6:** Coefficients of the best 5 °C model (based on AIC) for addition analysis with qWg corrected to 5 °C

Variable	Estimate	SE	*t*	*P*
5 °C model *R*^2^=79.8				
Intercept	110.3	8.45	13.05	<0.0001
Mg	−2 × 10^4^	4 × 10^5^	−4.40	<0.001
log_10_(qWg)	9.5	2.73	3.48	<0.005
Light.l (Low)	−41.2	4.78	−8.62	<0.0001
	Mode	Lower 95% CI		Upper 95% CI
Lambda (λ)	0	0		0.21

Mg = body mass (g); qWg = temperature-corrected (5 °C) mass-specific resting metabolic rate W/g; light.l = effect of low light levels on CFF in comparison to high light levels.

**Table A7:** Coefficients of the best 35 °C model (based on AIC) for both the main analysis and each of the sensitivity analyses

Variable	Estimate	SE	*t*	*P*
35 °C model *R*^2^=0.78.2				
Intercept	122.5	13.7	8.97	<0.0001
Mg	−2 × 10^4^	4 × 10^5^	−4.72	<0.0001
log_10_(qWg)	15.23	5.11	2.98	<0.01
Light.l (Low)	−41.47	4.99	−8.30	<0.0001
	Mode	Lower 95% CI		Upper 95% CI
Lambda (λ)	0	0		0.25

Mg = body mass (g); qWg = temperature-corrected (25 °C in main analysis) mass-specific resting metabolic rate W/g; light.l = effect of low light levels on CFF in comparison to high light levels.

**Table A8:** Coefficients of the model including brain mass using reduced data set of *N* = 28

Variable	Estimate	SE	*t*	*P*
Brain model *R*^2^=0.78				
Intercept	122.0	13.22	9.23	<0.0001
Mg	2 × 10^4^	4 × 10^5^	−4.33	<0.001
log_10_(qWg)	14.05	4.82	2.91	<0.01
Light.l (Low)	−43.02	5.60	−7.69	<0.0001
Brain mass	−0.005	0.01	−0.49	0.63
	Mode	Lower 95% CI		Upper 95% CI
Lambda (λ)	0	0		0.30

Mg = body mass (g); qWg = temperature-corrected (25 °C in main analysis) mass-specific resting metabolic rate W/g; light.l = effect of low light levels on CFF in comparison to high light levels; brain mass (g).

**Table A9:** Coefficients of the model including optimality of methodology factor

Variable	Estimate	SE	*t*	*P*
Model including optimality of study methodology *R*^2^=0.79				
Intercept	124.59	11.6	10.7	<0.0001
Mg	−2 × 10^4^	4 × 10^5^	−4.92	<0.001
log_10_(qWg)	13.8	3.94	3.50	<0.005
Light.l (Low)	−43.1	4.89	−8.81	<0.0001
Method (optimal)	−7.68	4.917	−1.56	0.13
	Mode	Lower 95% CI		Upper 95% CI
Lambda (λ)	0	0		0.22

Mg = body mass (g); qWg = temperature-corrected (25 °C in main analysis) mass-specific resting metabolic rate W/g; light.l = effect of low light levels on CFF; method = optimality of study methodology.

**Table 1:** Data set used in main analysis

Species	CFF	Mg	qWg	Brain mass	Light levels
*Ambystoma tigrinum*	30^e,s,1^	10.78^28^	0.00016^28^	NA	L
*Anguilla anguilla*	14^b,s,2^	71.1^28^	0.00013^28^	NA	L
*Anolis cristatellus*	70^e,o,3^	6.0^29^	0.00089^29^	NA	H
*Asio flammeus*	70^e,o,4^	406.0^30^	0.0032^28^	5.45^69^	H
*Bubo virginianus*	45^e,s,5^	1450.0^31^	0.0036^28^	13.7^70^	L
*Canis lupus familiaris*	80^b,s,6^	13900.0^32^	0.00183^28^	80.0^71^	H
*Carassius auratus*	67.2^e,o,7^	10.8^33^	0.00013^28^	0.01^71^	H
*Carcharhinus acronotus*	18^e,o,8^	14491.0^8^	0.00114^56^∗	NA	L
*Caretta caretta*	40^e,s,9^	135000.0^34^	0.00008^57^	2.7^40^	H
*Cavia porcellus*	50^e,s,10^	629.0^35^	0.00306^35^	3.8^72^	L
*Chelonia mydas*	40^e,s,9^	128000.0^36^	0.00025^36^	8.6^71^	H
*Columba livia*	100^e,s,4^	315.0^37^	0.0045^28^	2.3^70^	H
*Dermochelys coriacea*	15^e,s,11^	354000.0^38^	0.00043^58^	30.0^73^	H
*Felis catus*	55^e,s,12^	3054.4^32^	0.00394^59^	28.4^71^	L
*Gallus gallus domesticus*	87^b,o,13^	2710.0^39^	0.0022^28^	3.6^74^	H
*Gekko gecko*	20^e,s,14^	54.8^40^	0.00034^28^	0.2^75^	L
*Homo sapiens*	60^b,o,15^	67100.0^41^	0.00117^60^	1300.0^76^	H
*Iguana iguana*	80^e,s,14^	750.0^42^	0.00029^28^	0.61^75^	H
*Macaca mulatta*	95^b,o,16^	7710.0^43^	0.00205^61^	91.7^71^	H
*Melopsittacus undulatus*	74.7^b,s,17^	33.6^28^	0.01204^28^	1.5^70^	H
*Negaprion brevirostris*	37^e,s,18^	92987.0^44^	0.00053^62^∗	NA	L
*Oncorhynchus mykiss*	27^b,s,19^	4000.0^45^	0.00041^28^	0.5^71^	L
*Oryzias latipes*	37.2^e,s,20^	0.21^20^	0.00072^28^	0.01^77^	L
*Pagophilus groenlandicus*	32.7^b,s,12^	119600.0^46^	0.00211^63^	228.5^78^	L
*Raja erinacea*	30^e,o,22^	500.0^47^	0.00024^47^	2.32^71^	L
*Rattus norvegicus*	39^e,o,23^	237.0^48^	0.00679^48^	2.3^79^	L
*Spermophilus lateralis*	120^e,o,10^	215.5^49^	0.00335^64^	3.6^80^	H
*Sphenodon punctatus*	45.6^b,s,24^	353.75^50^	0.00017^28^	NA	L
*Sphyrna lewini*	27.3^e,o,8^	1893.0^8, 51^	0.0010^65^∗	60.0^77^	L
*Sturnus vulgaris*	100^e,s,25^	75.0^28^	0.012^28^	1.9^74^	H
*Tamias amoenus*	100^e,o,10^	51.91^52^	0.00937^66^	1.98^80^	H
*Tamiasciurus hudsonicus*	60^e,o,10^	215^35^	0.00735^67^	4.0^80^	H
*Thunnus albacares*	80^e,s,26^	45349.0^53, 54^	0.00158^68^∗	6.24^77^	H
*Tupaia glis*	90^b,o,27^	142.0^55^	0.00424^55^	3.4^79^	H

CFF = critical flicker fusion; Mg = body mass (g); qWg = temperature-corrected (25 °C) mass-specific resting metabolic rate (W/g); light levels: H = high, L = low; NA = no data available for species. Superscript indicates type of measurement: e = electroretinogram; b = behavioural experiments; o = optimum methodology; s = suboptimum methodology; numbers refer to data sources: (1) Crevier & Meister (1998); (2) Adrian & Matthews (1926); (3) Fleishman et al. (1995); (4) Bornshein & Tansley (1961); (5) Ault & House (1987); (6) Coile et al. (1989); (7) Hanyu & Ali (1963); (8) McComb et al. (2010); (9) Levenson et al. (2004); (10) Tansley et al. (1961); (11) Eckert et al. (2006); (12) Loop & Berkeley (1975); (13) Lisney et al. (2011); (14) Meneghini & Hamasaki (1967); (15) Brundrett (1974); (16) Shumake et al. (1968); (17) Ginsburg & Nilsson (1971); (18) Gruber (1969); (19) Carvalho et al. (2004); (20) Carvalho et al. (2002); (21) Bernholz & Matthews (1975); (22) Green & Siegel (1975); (23) Williams et al. (1985); (24) Woo et al. (2009); (25) Greenwood et al. (2004); (26) Southwood et al. (2008); (27) Callahan & Petry (1999); (28) Makarieva et al. (2008); (29) Rogowitz (1996); (30) Graber (1962); (31) Ganey et al. (1993); (32) Kendall et al. (1982); (33) Hughes (1977); (34) Duermit (2007); (35) Arends & McNab (2001); (36) Jackson & Prange (1979); (37) Terres (1980); (38) Georges & Fossette (2006); (39) Winchester (1940); (40) Hurlburt (1996); (41) Holloway (1980); (42) Howland et al. (2004); (43) Schwartz & Kemnitz (1992); (44) Allyn (1947); (45) Ridolfi (2006); (46) Stewart & Lavigne (1984); (47) Hove & Moss (1997); (48) Hart (1971); (49) McKeever (1964); (50) Herrel et al. (2010); (51) Letourneur et al. (1998); (52) Sheppard (1968); (53) Collette & Nauen (1983); (54) Duarte-Neto & Lessa (2004); (55) Bradley & Hudson (2003); (56) Carlson et al. (1999); (57) Lutz et al. (1989); (58) Paladino et al. (1996); (59) Eisenberg (1981); (60) Elgar & Harvey (1987); (61) Bruhn (1934); (62) Bushnell et al. (1989); (63) McNab (1986); (64) Hudson et al. (1972); (65) Lowe (2001); (66) Jones & Wang (1976); (67) Pauls (1981); (68) Dewar & Graham (1994); (69) Garamszegi et al. (2002); (70) Iwaniuk & Nelson (2002); (71) Crile & Quiring (1940); (72) Herculano-Houzel et al. (2006); (73) Davenport et al. (2009); (74) Burton (2008); (75) Platel (1979); (76) Aiello & Wheeler (1995); (77) Froese & Pauly (2012); (78) Walløe et al. (2010); (79) Navarret et al. (2011); (80) Meier (1983).

∗ Indicates species with qWg estimated from swimming speeds extrapolated to zero (see Methods).

**Table 2:** Coefficients of the two most parsimonious models in the main analysis (based on AIC)

Variable	Estimate	SE	*t*	*P*
Model 1 *R*^2^=0.79				AIC=275.70
Intercept	118.60	11.30	10.54	<0.0001
Mg	−2 × 10^4^	4 × 10^5^	−4.45	<0.001
log_10_(qWg)	13.20	4.02	3.30	<0.005
Light.l (low)	−41.12	4.87	−8.44	<0.0001
	Mode	Lower 95% CI		Upper 95% CI
Lambda (λ)	0	0		0.22
Model 2 *R*^2^=0.78				AIC=277.68
Intercept	118.90	12.00	9.94	<0.0001
Mg	−2 × 10^4^	4 × 10^5^	−4.45	<0.001
log_10_(qWg)	13.24	4.08	3.24	<0.005
Light.l (low)	−41.10	4.96	−8.28	<0.0001
Exp.t (ERG)	−0.51	5.08	−0.10	0.92
	Mode	Lower 95% CI		Upper 95% CI
Lambda (λ)	0	0		0.22

Mg = body mass (g); qWg = temperature-corrected (25 °C in main analysis) mass-specific resting metabolic rate W/g; light.l (low) = effect of low light levels on CFF in comparison to high light levels and exp.t = effect of experimental type (ERG = electroretinogram) in comparison to behaviour-based CFF measures.

## Appendix

### Phylogeny Reconstruction

We used divergence times and phylogenies from the literature to produce a composite phylogeny of the vertebrate species used in our analyses (Fig. A1). For species with no available divergence dates based on molecular data or available published trees, we used conservatively estimated first appearance dates from the Paleobiology Database as an estimate of divergence time (Alroy et al. 2008).

We took divergence dates for the major groups Elasmobranchii, Actinopterygii and Amphibia from the TimeTree database (Hedges et al. 2006). For divergence dates of Carcharhinidae and Sphyrnidae we used estimates from Lim et al. (2010), while the divergence time between *Negaprion brevirostris* and *Carcharhinus acronotus* was estimated based on the appearance of *N. brevirostris*, the younger of the two species (*Negaprion* spp.: 40.3 million years ago; *Carcharhinus*: 46.2 million years ago). For Actinopterygii we used Li et al. (2008) to infer their phylogenetic relationships and divergence times, and Little et al. (2010) for Perciformes' divergence times. We used Benton & Donoghue's (2007) estimation of divergence time between anopsids (Testudines and Aves) and suarapids (Squamata and Rhynchocephalia) while Perelman et al. (2011) was used for the divergence and phylogenetic relationships among Squamata, Rhynchocephalia, Testudines and Aves. For divergence times within the Squamata we used Wiens et al. (2006), while for Testudines we used Naro-Maciel et al. (2008). We used Brown et al. (2008) for the Aves phylogeny with divergence times between *Asio flammeus* and *Bubo virginianus* estimated from the first appearance in the fossil record (Janossy 1986). We used Murphy et al. (2007) for divergence dates of Mammalia orders, while for Primates we used Perelman et al. (2011). Rodentia divergence times were taken from Murphy et al. (2007). All references relating to the phylogeny are given in the reference list.

### Sensitivity Analyses

We performed a series of sensitivity analyses to test whether the results of our main analysis were affected by (1) the temperature to which ectoderm species' metabolic rates were corrected, (2) the inclusion of brain mass as a control for information-processing abilities and (3) the quality of the data used in the analysis.

(1) Ectotherm temperature. We used Q10 values, the fold change in metabolic rate over a temperature change of 10 °C, as defined for each of the major groups (i.e. reptilian, amphibian, etc.; see Methods), to correct ectotherm mass-specific metabolism (qWg) over a temperature range of 5 °C–35 °C. We performed this analysis by rerunning the main analysis with qWg corrected to 5 °C and then corrected to 35 °C. The resulting set of models and the terms that they included are given in Tables A2 and A3. In both analyses the model with the lowest AIC includes the same terms as found in the main analysis, i.e. body mass (Mg), temperature-corrected mass-specific resting metabolic rate (qWg) and light levels, with qualitatively the same significant effects (Fig. A2, Tables A6 and A7).

(2) Brain mass. As the amount of sensory tissue available to an organism may aid in its ability to perceive and process information, brain mass values, measured as wet weight (g), were taken from the literature (Table 1, Methods). As data on brain mass were available for only a subset of 28 species, we included the term brain mass along with the terms used in the main analysis (light levels, qWg, experimental design and body mass) in a series of models performed on the restricted data set (Table A4). While we found a similar trend to the first analysis with a positive effect of log_10_ mass-specific resting metabolic rate (14.05 ± 4.82) and negative effects of low light levels (−43.02 ± 5.6) and body mass (−0.0002 ± 0.00004), brain mass was found to have no significant effect on CFF levels (Table A8).

(3) Optimality of study methodology for measuring maximum CFF. To ensure that data quality did not affect the results of our analysis we coded the values from each study as either methodologically optimum or methodologically suboptimum. Only data from studies that used a sufficient range of flicker and light intensity within the experimental procedure were coded as methodologically optimum (Table 1, Methods).

To test the possible effect relating to this grouping on our results we reran the main analysis with these categories added as a fixed factor. The resulting set of terms included in the top models are given in Table A5. In the best model based on AIC that includes the methodology optimality term, all terms found in the main analysis (light levels, log_10_ of qWg and body mass) were found to be included. As in the main results Mg and light levels had significantly negative effects, and log_10_(qWg) had a significantly positive effect and the methodology optimality term had no significant effect on CFF (Table A9).

As methodologically suboptimum data would be expected to give values below the maximum CFF and hence produce negative residuals in our models, we also performed a Mann–Whitney *U* test between residuals taken from the main model representing both methodology categories (Table 2, Results in main text). We found no significance difference between the residuals representing methodologically optimum and suboptimum data and also visually found an even spread of each type of residual when plotted (Mann–Whitney *U* test: *U* = 185, *N*_s_ = 20, *N*_o_ = 14, *P* = 0.09; Figs A3 and A4).

## References

[bib1] Adrian E.D., Matthews R. (1926). The action of light on the eye. Part III. The interaction of retinal neurons. Journal of Physiology.

[bib2] Aiello L.C., Wheeler P. (1995). The expensive-tissue hypothesis. Current Anthropology.

[bib3] Albert E.M., Mauro D.S., García-París M., Rüber L., Zardoya R. (2009). Effect of taxon sampling on recovering the phylogeny of squamate reptiles based on complete mitochondrial genome and nuclear gene sequence data. Gene.

[bib4] Ali M.A., Klyne M.A. (1985). Vision in Vertebrates.

[bib5] Allyn R. (1947). A Dictionary of Fishes.

[bib6] Alroy J., Aberhan M., Bottjer D.J., Foote M., Fürsich F.T., Harries P.J., Hendy A.J., Holland S.M., Ivany L.C., Kiessling W. (2008). Phanerozoic trends in the global diversity of marine invertebrates. Science.

[bib7] Arends A., McNab B.K. (2001). The comparative energetics of ‘caviomorph’ rodents. Comparative Biochemistry and Physiology Part A: Molecular & Integrative Physiology.

[bib8] Ault S.J., House E.W. (1987). Electroretinographic responses of the great horned owl (*Bubo virginianus*). Journal of Raptor Research.

[bib9] Autrum H. (1958). Electrophysiological analysis of the visual systems in insects. Experimental Cell Research.

[bib10] Bahill T., Baldwin D.G., Hung G., Pallis J. (2004). The rising fastball and other perceptual illusions of batters. Biomedical Engineering Principles in Sports.

[bib11] Benton M.J., Donoghue P.C.J. (2007). Paleontological evidence to date the tree of life. Molecular Biology and Evolution.

[bib12] Bernholz C.D., Matthews M.L. (1975). Critical flicker frequency in a harp seal: evidence for duplex retinal organization. Vision Research.

[bib13] Bornshein H., Tansley K. (1961). Elektroretinogramm und netzhautstruktur der Sumpfohreule (*Asio flammeus*). Experientia.

[bib14] Biewener A.A. (2003). Animal Locomotion.

[bib15] Block B.A., Carey F.G. (1985). Warm brain and eye temperatures in sharks. Journal of Comparative Physiology B.

[bib16] Bradley S.R., Hudson J.W. (2003). Temperature regulation in the tree shrew *Tupaia glis*. Comparative Biochemistry and Physiology Part A: Physiology.

[bib17] Brown J.H., Gillooly J.F., Allen A.P., Savage V.M., West G.B. (2004). Toward a metabolic theory of ecology. Ecology.

[bib18] Brown J.W., Rest J.S., García-Moreno J., Sorenson M.D., Mindell D.P. (2008). Strong mitochondrial DNA support for a Cretaceous origin of modern avian lineages. BMC Biology.

[bib19] Bruhn J.M. (1934). The respiratory metabolism of infra-human primates. American Journal of Physiology.

[bib20] Brundrett G.W. (1974). Human sensitivity to flicker. Lighting Research and Technology.

[bib21] Burnham K.P., Anderson D.R. (2002). Model Selection and Multimodel Inference: a Practical Information – Theoretic Approach.

[bib22] Burton R.F. (2008). The scaling of eye size in adult birds: relationship to brain, head and body sizes. Vision Research.

[bib23] Bushnell P.G., Lutz P.L., Gruber S.H. (1989). The metabolic rate of an active, tropical elasmobranch, the lemon shark (*Negaprion brevirostris*). Journal of Experimental Biology.

[bib24] Carey F.G. (1982). A brain heater in the swordfish. Science.

[bib25] Callahan T.L., Petry H.M. (1999). Psychophysical measurement of temporal modulation sensitivity in the tree shrew (*Tupaia belangeri*). Vision Research.

[bib26] Carlson J.K., Palmer C.L., Parsons G.R. (1999). Oxygen consumption rate and swimming efficiency of the blacknose shark, *Carcharhinus acronotus*. Copeia.

[bib27] Carvalho P.S.M., Noltie D.B., Tillitt D.E. (2002). Ontogenetic improvement of visual function in the medaka *Oryzias latipes* based on an optomotor testing system for larva and adult fish. Animal Behaviour.

[bib28] Carvalho P.S.M., Noltie D.B., Tillitt D.E. (2004). Biochemical, histological and behavioural aspects of visual functioning during early development of rainbow trout. Journal of Fish Biology.

[bib29] Clark R.W., Tangco S., Barbour M.A. (2012). Field video recordings reveal factors influencing predatory strike success of free-ranging rattlesnakes (*Crotalus* spp.). Animal Behaviour.

[bib30] Coile D.C., Pollitz C.H., Smith J.C. (1989). Behavioral determination of critical flicker fusion in dogs. Physiology and Behavior.

[bib31] Collette B.B., Nauen C.E. (1983). FAO Species Catalogue.

[bib32] Crevier D.W., Meister M. (1998). Synchronous period-doubling in flicker vision in salamander and man. Journal of Neurophysiology.

[bib33] Crile G., Quiring D.P. (1940). A record of the body weight and certain organ and gland weights of 3690 animals. The Ohio Journal of Science.

[bib34] Cronin T.W., Barbosa P., Castellanos I. (2005). The role of vision in predator–prey interactions. Ecology of Predator–Prey Interactions.

[bib35] Davenport J., Fraher J., Fitzgerald E., McLaughlin P., Doyle T., Harman L., Cuffe T. (2009). Fat head: an analysis of head and neck insulation in the leatherback turtle (*Dermochelys coriacea*). Journal of Experimental Biology.

[bib36] D'Eath R.B. (1998). Can video images imitate real stimuli in animal behavior experiments?. Biological Reviews.

[bib37] De Vries S.E.J., Clandinin T.R. (2012). Loom-sensitive neurons link computation to action in the *Drosophila* visual system. Current Biology.

[bib38] Dewar H., Graham J.B. (1994). Studies of tropical tuna swimming performance in a large water tunnel. Journal of Experimental Biology.

[bib39] Dessing J.C., Craig C.M. (2010). Bending it like Beckham: how to visually fool the goalkeeper. PLoS One.

[bib40] Duarte-Neto P.J., Lessa R.P., Lessa R.P., Nóbrega M.F., Júnior J.L.B. (2004). Thunnus albacares. Dinâmica de Populações e Avaliação de Estoques dos Recursos Pesqueiros da Região Nordeste.

[bib41] Dudley R. (2002). Mechanism and implications of animal maneuverability. Integrative and Comparative Biology.

[bib42] Duermit L. (2007). ‘*Caretta caretta*’ Animal Diversity Web. http://animaldiversity.ummz.umich.edu/site/accounts/information/Caretta_caretta.html.

[bib43] Eckert S., Levenson D., Crognale M., Swimmer Y., Brill R. (2006). The sensory biology of sea turtles: what can they see, and how can this help them avoid fishing gear?. Sea Turtle and Pelagic Fish Sensory Biology: Developing Techniques to Reduce Sea Turtle Bycatch in Longline Fisheries.

[bib44] Eisenberg J.F. (1981). The Mammalian Radiations.

[bib45] Elgar M.A., Harvey P.H. (1987). Basal metabolic rates in mammals: allometry, phylogeny and ecology. Functional Ecology.

[bib46] Ferry F.S. (1892). Persistence of vision. American Journal of Science.

[bib47] Fleishman L.J., Marshall C.J., Hertz P.E. (1995). Comparative study of temporal response properties of the visual system of three species of anoline lizards. Copeia.

[bib48] Folkow L.P., Nordoy E.S., Blix A.S. (2004). Distribution and diving behaviour of harp seals (*Pagophilus groenlandicus*) from the Greenland Sea stock. Polar Biology.

[bib49] Frank T.M. (1999). Comparative study of temporal resolution in the visual system of mesopelagic crustaceans. Biology Bulletin.

[bib50] Frank T.M., Johnsen S., Cronin T.W. (2012). Light and vision in the deep-sea benthos: II. Vision in deep-sea crustaceans. Journal of Experimental Biology.

[bib51] Franz A., Ronacher B. (2002). Temperature dependence of temporal resolution in an insect nervous system. Journal of Comparative Physiology A.

[bib52] Freckleton R.P. (2009). The seven deadly sins of comparative analysis. Journal of Evolutionary Biology.

[bib53] Fritsches K.A., Brill R.W., Warrant E.J. (2005). Warm eyes provide superior vision in swordfishes. Current Biology.

[bib54] Froese R., Pauly D. (2012). FishBase. http://www.fishbase.org.

[bib55] Ganey J.L., Balda R.P., King R.M. (1993). Metabolic rate and evaporative water loss of Mexican spotted and great horned owls. Wilson Bulletin.

[bib56] Garamszegi L.Z., Møller A.P., Erritzøe J. (2002). Coevolving avian eye size and brain size in relation to prey capture and nocturnality. Proceedings of the Royal Society B.

[bib57] Georges J.Y., Fossette S. (2006). Estimating body mass in leatherback turtles *Dermochelys coriacea*. Marine Ecology Progress Series.

[bib58] Gilbert C. (1997). Visual control of cursorial prey pursuit by tiger beetles (*Cicindelidae*). Journal of Comparative Physiology A.

[bib59] Ginsburg N., Nilsson V. (1971). Measuring flicker thresholds in the budgerigar. Journal of Experimental Analysis of Behavior.

[bib60] Graber R.R. (1962). Food and oxygen consumption in three species of owls (*Strigidae*). The Condor.

[bib61] Green D.G., Siegel I.M. (1975). Double branched flicker fusion curves from the all-rod skate retina. Science.

[bib62] Greenwood V.J., Smith E.L., Goldsmith A.R., Cuthill I.C., Crisp L.H., Walter-Swan M.B., Bennett A.T.D. (2004). Does the flicker frequency of fluorescent lighting affect the welfare of captive European starlings?. Applied Animal Behaviour Science.

[bib63] Gruber S.H. (1969). The physiology of vision in the lemon shark, Negaprion brevirostris (Poey): a behavioral analysis.

[bib64] Haddock S.H.D., Dunn C.W., Pugh P.R., Schnitzler C.E. (2005). Bioluminescent and red-fluorescent lures in a deep-sea siphonophore. Science.

[bib65] Hagura N., Kanai R., Orgs G., Haggard P. (2012). Ready steady slow: action preparation slows the subjective passage of time. Proceedings of the Royal Society B.

[bib66] Hanyu I., Ali M.A. (1963). Flicker fusion frequency of electroretinogram in light-adapted goldfish at various temperatures. Science.

[bib67] Hart J.S., Whittow G.G. (1971). Rodents. Comparative Physiology of Thermoregulation.

[bib68] Hedges S.B., Dudley J., Kumar S. (2006). TimeTree: a public knowledge-base of divergence times among organisms. Bioinformatics.

[bib69] Hedrick T.L. (2011). Damping in flapping flight and its implications for manoeuvring, scaling and evolution. Journal of Experimental Biology.

[bib70] Heglund N.C., Taylor C.R. (1988). Speed, stride frequency and energy cost per stride: how do they change with body size and gait?. Journal of Experimental Biology.

[bib71] Herculano-Houzel S., Mota B., Lent R. (2006). Cellular scaling rules for rodent brains. Proceedings of the National Academy of Sciences, U.S.A..

[bib72] Herrel A., Moore J.A., Bredeweg E.M., Nelson N.J. (2010). Sexual dimorphism, body size, bite force and male mating success in tuatara. Biological Journal of the Linnean Society.

[bib73] Holloway R.L. (1980). Within species brain-body weight variability: a re-examination of the Danish data and other primate species. American Journal of Physical Anthropology.

[bib74] Hornstein E.P., O'Carroll D.C., Anderson J.C., Laughlin S.B. (2000). Sexual dimorphism matches photoreceptor performance to behavioural requirements. Proceedings of the Royal Society B.

[bib75] Hove J.R., Moss S.A. (1997). Effect of MS-222 on response to light and rate of metabolism of the little skate *Raja erinacea*. Marine Biology.

[bib76] Howland H.C., Merola S., Basarab J.R. (2004). The allometry and scaling of the size of vertebrate eyes. Vision Research.

[bib77] Hudson J.W., Deavers D.R., Bradley S.R. (1972). A comparative study of temperature regulation in ground squirrels with special reference to the desert species. Symposia of the Zoological Society of London.

[bib78] Hughes A., Crescitelli F. (1977). The topography of vision in mammals of contrasting life style: comparative optics and retinal organisation. Handbook of Sensory Physiology, VII/5: The Visual System in Vertebrates.

[bib79] Hurlburt G.R. (1996). Relative brain size in recent and fossil amniotes: determination and interpretation.

[bib80] Iwaniuk A.N., Nelson J.E. (2002). Can endocranial volume be used as an estimate of brain size in birds. Canadian Journal of Zoology.

[bib81] Jackson D.C., Prange H.D. (1979). Ventilation and gas exchange during rest and exercise in adult green sea turtles. Journal of Comparative Physiology A.

[bib82] Janossy D. (1986). Pleistocene Vertebrate Faunas of Hungary.

[bib83] Jones D.L., Wang L.C.H. (1976). Metabolic and cardiovascular adaptations in the western chipmunks, genus *Eutamias*. Journal of Comparative Physiology.

[bib84] Kendall P.T., Blaza S.E., Smith P.M. (1982). Comparative digestible energy requirements of adult beagles and domestic cats for body weight maintenance. Journal of Nutrition.

[bib85] Kreysing M., Pusch R., Haverkate D., Landsberger M., Engelmann J., Ruiter J., Mora-Ferrer C., Ulbricht E., Grosche J., Franze K. (2012). Photonic crystal light collectors in fish retina improve vision in turbid waters. Science.

[bib86] Land M.F., Collett T.S. (1974). Chasing behavior of houseflies (*Fannia canicularis*): description and analysis. Journal of Comparative Physiology A.

[bib87] Laughlin S.B. (2001). Energy as a constraint on the coding and processing of sensory information. Current Opinion in Neurobiology.

[bib88] Laughlin S.B., Weckström M. (1993). Fast and slow photoreceptors a comparative-study of the functional diversity of coding and conductances in the diptera. Journal of Comparative Physiology A.

[bib89] Letourneur Y., Kulbicki M., Labrosse P. (1998). Length-weight relationships of fish from coral reefs and lagoons of New Caledonia, southwestern Pacific Ocean: an update. Naga: International Centre for Living Aquatic Resources Management Quarterly.

[bib90] Levenson D., Eckert S., Crognale M., Deegan J.I., Jacobs G. (2004). Photopic spectral sensitivity of green and loggerhead sea turtles. Copeia.

[bib175] Li C., Lu G., Ortí G. (2008). Optimal data partitioning and a test case for ray-finned fishes (Actinopterygii) based on ten nuclear loci. Systematic Biology.

[bib91] Lim D.D., Motta P., Mara K., Martin A.P. (2010). Phylogeny of hammerhead sharks (Family Sphyrnidae) inferred from mitochondrial and nuclear genes. Molecular Phylogenetics and Evolution.

[bib92] Lisney T.J., Rubene D., Rózsa J., Lølie H., Håstad O., Ödeen A. (2011). Behavioural assessment of flicker fusion frequency in chicken *Gallus gallus domesticus*. Vision Research.

[bib93] Little A.G., Lougheed S.C., Moyes C.D. (2010). Evolutionary affinity of billfishes (*Xiphiidae* and *Istiophoridae*) and flatfishes (*Plueronectiformes*): independent and trans-subordinal origins of endothermy in teleost fishes. Molecular Phylogenetics and Evolution.

[bib94] Loop M.S., Berkeley M.S. (1975). Temporal modulation sensitivity of the cat: I. Behavioral methods. Vision Research.

[bib95] Lowe C.G. (2001). Metabolic rates of juvenile scalloped hammerhead sharks (*Sphyrna lewini*). Marine Biology.

[bib96] Lutz P.L., Bergey A., Bergey M. (1989). Effects of temperature on gas exchange and acid–base balance in the sea turtle *Caretta caretta* at rest and during routine activity. Journal of Experimental Biology.

[bib97] McComb D.M., Frank T.M., Hueter R.E., Kajiura S.M. (2010). Temporal resolution and spectral sensitivity of the visual system of three coastal shark species from different light environments. Physiological and Biochemical Zoology.

[bib98] McGill B.J., Mittelbach G.G. (2006). An allometric vision and motion model to predict prey encounter rates. Evolutionary Ecology Research.

[bib99] McKeever S. (1964). The biology of the golden-mantled ground squirrel, *Citellus lateralis*. Ecological Monographs.

[bib100] McNab B.K. (1986). The influence of food habits on the energetics of eutherian mammals. Ecological Monographs.

[bib101] Makarieva A.M., Gorshkov V.G., Li B.L., Chown S.L., Reich P.B., Gavrilov V.M. (2008). Mean mass-specific metabolic rates are strikingly similar across life's major domains: evidence for life's metabolic optimum. Proceedings of the National Academy of Sciences, U.S.A..

[bib102] Meier P.T. (1983). Relative brain size within the North American Sciuridae. Journal of Mammalogy.

[bib103] Meneghini K.A., Hamasaki D.I. (1967). The electroretinogram of the iguana and Tokay gecko. Vision Research.

[bib104] Mercer J.M., Roth L. (2003). The effects of Cenozoic global change on squirrel phylogeny. Science.

[bib105] Murphy W.J., Pringle T.H., Crider T.A., Springer M.S., Miller W. (2007). Using genomic data to unravel the root of the placental mammal phylogeny. Genome Research.

[bib106] Myers P., Espinosa R., Parr C.S., Jones T., Hammond G.S., Dewey T.A. (2012). The Animal Diversity Web. http://animaldiversity.org.

[bib107] Naro-Maciel E., Le M., FitzSimmons N.N., Amato G. (2008). Evolutionary relationships of marine turtles: a molecular phylogeny based on nuclear and mitochondrial genes. Molecular Phylogenetics and Evolution.

[bib108] Navarret A., van Schaik C.P., Isler K. (2011). Energetics and the evolution of human brain size. Nature.

[bib109] Orme C.D.L., Freckleton R.P., Thomas G.H., Petzoldt T., Fritz S.A., Isaac N.J.B., Pearse W. (2012). Caper: comparative analysis of phylogenetics and evolution in R. Methods in Ecology and Evolution.

[bib110] Pagel M. (1999). Inferring the historical patterns of biological evolution. Nature.

[bib111] Paladino F.V., Spotila J.R., O'Connor M.P., Gatten R.E. (1996). Respiratory physiology of adult leatherback turtles (*Dermochelys coriacea*) while nesting on land. Chelonian Conservation and Biology.

[bib112] Palmer J.M., Grant B.G. (2010). The Art of Radiometry.

[bib113] Pauls R.W. (1981). Energetics of the red squirrel: a laboratory study of the effects of temperature, seasonal acclimatization, use of the nest and exercise. Journal of Thermal Biology.

[bib114] Pawar S., Dell A.I., Savage V.M. (2012). Dimensionality of consumer search space drives trophic interaction strengths. Nature.

[bib115] Perelman P., Johnson W.E., Roos C., Seuánez H.N., Horvath J.E., Moreira M.A.M., Kessing B., Pontius J., Roelke M., Rumpler Y. (2011). A molecular phylogeny of living primates. PLoS Genetics.

[bib116] Platel R., Gans C., Northcutt R.G., Ulinski P. (1979). Brain weight-body weight relationships. Biology of the Reptilia. Vol. 9.

[bib117] Porter T.C. (1902). Contributions to the study of flicker. Proceedings of the Royal Society B.

[bib118] R Development Core Team (2012). R: a Language and Environment for Statistical Computing.

[bib119] Ridolfi K. (2006). ‘*Oncorhynchus mykiss*’. Animal Diversity Web. http://animaldiversity.ummz.umich.edu/site/accounts/information/Oncorhynchus_mykiss.html.

[bib120] Rogowitz G.L. (1996). Evaluation of thermal acclimation of metabolism in two eurythermal lizards, *Anolis cristatellus* and *A. sagrei*. Journal of Thermal Biology.

[bib121] Rohlf F.J. (2001). Comparative methods for the analysis of continuous variables: geometric interpretations. Evolution.

[bib122] Rubene D., Håstad O., Tauson R., Wall H., Ödeen A. (2010). The presence of UV wavelengths improves the temporal resolution of the avian visual system. Journal of Experimental Biology.

[bib123] Sato K., Watanuki Y., Takahashi A., Miller P.J.O., Tanaka H., Kawabe R., Ponganis P.J., Handrich Y., Akamatsu T., Watanabe Y. (2007). Stroke frequency, but not swimming speed, is related to body size in free-ranging seabirds, pinnipeds and cetaceans. Proceedings of the Royal Society B.

[bib124] Schoch R.M. (1985). Preliminary description of a new late Paleocene land-mammal fauna from South Carolina, U.S.A. Postilla.

[bib125] Schwartz S.H. (2009). Visual Perception: a Clinical Orientation.

[bib126] Schwartz S.M., Kemnitz J.W. (1992). Age- and gender-related changes in body size, adiposity, and endocrine and metabolic parameters in free-ranging rhesus macaques. American Journal of Physical Anthropology.

[bib127] Sheppard D.H. (1968). Seasonal changes in body and adrenal weights of chipmunks (*Eutamias*). Journal of Mammalogy.

[bib128] Shumake S.A., Smith J.C., Taylor H.L. (1968). Critical fusion frequency in rhesus monkeys. The Psychological Record.

[bib129] Sibly R.M., Brown J.H., Kodric-Brown A. (2012). Metabolic Ecology, a Scaling Approach.

[bib130] Southwood A., Fritsches K., Brill R., Swimmer Y. (2008). Sound, chemical, and light detection in sea turtles and pelagic fishes: sensory-based approaches to bycatch reduction in longline fisheries. Endangered Species Research.

[bib131] Stevens M. (2007). Predator perception and the interrelation between different forms of protective coloration. Proceedings of the Royal Society B.

[bib132] Stevens M., Searle W.T.L., Seymour J.E., Marshall K.L.A., Ruxton G.D. (2011). Motion dazzle and camouflage as distinct anti-predator defences. BMC Biology.

[bib133] Stewart R.E.A., Lavigne D.M. (1984). Energy transfer and female condition in nursing harp seals *Phoca groenlandica*. Holarctic Ecology.

[bib134] Tansley K. (1957). Vision in Vertebrates.

[bib135] Tansley K., Copenhaver R.M., Gunkel R.D. (1961). Some aspects of the electroretinographic response of the American red squirrel, *Tamiasciurus hudsonicus loquax*. Journal of Cellular and Comparative Physiology.

[bib136] Tatler B., O'Carroll D.C., Laughlin S.B. (2000). Temperature and the temporal resolving power of fly photoreceptors. Journal of Comparative Physiology A.

[bib137] Terres J.K. (1980). The Audubon Society Encyclopedia of North American Birds.

[bib138] Vogel S. (2008). Modes and scaling in aquatic locomotion. Integrative and Comparative Biology.

[bib139] Walløe S., Eriksen N., Dabelsteen T., Pakkenberg B. (2010). A neurological comparative study of the harp seal (*Pagophilus groenlandicus*) and harbor porpoise (*Phocoena phocoena*) brain. The Anatomical Record.

[bib140] Watanabe Y.Y., Lydersen C., Fisk A.T., Kovacs K.M. (2012). The slowest fish: swim speed and tail-beat frequency of Greenland sharks. Journal of Experimental Marine Biology and Ecology.

[bib141] White C.R., Phillips N.F., Seymour R.S. (2006). The scaling and temperature dependence of vertebrate metabolism. Biology Letters.

[bib142] Widder E.A. (2010). Bioluminescence in the ocean: origins of biological, chemical, and ecological diversity. Science.

[bib143] Wiens J.J., Brandley M.C., Reeder T.W. (2006). Why does a trait evolve multiple times within a clade? Repeated evolution of snakelike body form in squamate reptiles. Evolution.

[bib144] Williams R.A., Pollitz C.H., Smith J.C., Williams T.P. (1985). Flicker detection in the albino rat following light-induced retinal damage. Physiology and Behaviour.

[bib145] Winchester C.F. (1940). Seasonal and Metabolic Rhythms in the Domestic Fowl.

[bib146] Woo K.L., Hunt M., Harper D., Nelson N.J., Daugherty C.H., Bell B.D. (2009). Discrimination of flicker fusion rates in the reptile tuatara (*Sphenodon*). Naturwissenschaften.

[bib147] Woodward G., Ebenman B., Emmerson M., Montoya J.M., Olesen J.M., Valido A., Warrenm P.H. (2005). Body size in ecological networks. Trends in Ecology & Evolution.

